# Impact of Driver Genetic Alterations on Survival in Metastatic Colorectal Cancer Patients from a Genetically Homogeneous Sardinian Population: A Real-World Study

**DOI:** 10.3390/cancers18111708

**Published:** 2026-05-23

**Authors:** Grazia Palomba, Luca Nuvoli, Maria Cristina Sini, Giovanni Battista Maestrale, Maria Grazia Doro, Laura Frogheri, Ivana Persico, Angelo Zinellu, Davide Adriano Santeufemia, Panagiotis Paliogiannis, Daniele Delogu, Fabrizio Scognamillo, Giuseppe Palmieri

**Affiliations:** 1 Unit of Cancer Genetics, Institute of Genetic Biomedical Research (IRGB), National Research Council (CNR), Traversa La Crucca 3, 07100 Sassari, Italy; grazia.palomba@cnr.it (G.P.); lucanuvoli@cnr.it (L.N.); mariacristina.sini@cnr.it (M.C.S.); giovannibattista.maestrale@cnr.it (G.B.M.); mariagrazia.doro@cnr.it (M.G.D.); marialaura.frogheri@cnr.it (L.F.); ivana.persico@cnr.it (I.P.); 2 University Hospital (AOU) of Sassari, Viale San Pietro 43, 07100 Sassari, Italy; ppaliogiannis@uniss.it (P.P.); daniele.delogu@aouss.it (D.D.); 3 Department of Biomedical Sciences, University of Sassari, Viale San Pietro 43, 07100 Sassari, Italy; azinellu@uniss.it; 4 Oncology Unit, Civil Hospital, Via Don Minzoni, 07041 Alghero, Italy; davideadriano.santeufemia@aslsassari.it; 5 Department of Medicine, Surgery and Pharmacy, University of Sassari, Viale San Pietro 43, 07100 Sassari, Italy; fscognamillo@uniss.it; 6 Immuno-Oncology & Targeted Cancer Biotherapies, University of Sassari, Viale San Pietro 43, 07100 Sassari, Italy

**Keywords:** colorectal cancer, mutation analysis, *KRAS*, *NRAS*, *BRAF*, MSI, prognosis

## Abstract

**Background**: Colorectal cancer (CRC) is the third most diagnosed malignancy and the second leading cause of cancer-related mortality worldwide. Recent therapeutic advancements have significantly improved clinical management, underscoring the importance of routine molecular profiling to guide personalised treatment strategies. This study aims to evaluate the prognostic impact of main molecular alterations—including allele frequency (AF) of RAS mutations—on survival outcomes in a real-world hospital-based cohort of patients with metastatic CRC. **Methods**: A total of 208 consecutive patients with a histologically confirmed diagnosis of CRC and complete clinical, molecular, and survival data were retrospectively analysed. Somatic mutations in *KRAS*, *NRAS*, *BRAF,* and the occurrence of microsatellite instability (MSI) were assessed using pyrosequencing and real-time PCR assays, respectively, on formalin-fixed, paraffin-embedded tumour samples. Associations between mutational status, clinicopathological parameters, and overall survival (OS) were evaluated. **Results**: Overall, 138 patients (66.3%) harboured at least one somatic mutation: 115 (55.3%) in *KRAS*, 8 (3.8%) in *NRAS*, and 15 (7.2%) in *BRAF*. MSI was detected in 17/208 (8.2%) patients. A statistically significant improvement in OS was observed in patients lacking mutations in any of the three genes—referred to as wild-type (WT) patients—with *BRAF* mutated cases showing the worst survival (*p* = 0.041). Increasing age at the time of first-line therapy for advanced disease stage was associated with a statistically significant increase in the hazard of death (*p* = 0.031). **Conclusions**: In the advanced disease stage, *RAS/BRAF* wild-type colorectal cancers were significantly associated with a survival advantage.

## Simple Summary

Colorectal cancer (CRC) ranks as the third most frequently diagnosed malignancy and the second leading cause of cancer mortality worldwide. This retrospective study analysed 208 patients with metastatic CRC, assessing *KRAS*, *NRAS*, and *BRAF* mutational status as well as microsatellite instability (MSI) occurrence. Mutually exclusive mutations were detected in 138 (66%) cases, predominantly in *KRAS* (55%). MSI was found in 17 (8%) cases. The median overall survival (OS) was higher (38 months) in wild-type patients as compared with patients with *RAS* (26 months) or *BRAF* (14 months) mutations. No significant OS differences emerged according to MSI status or variant allele frequency (AF) in mutated colorectal patients. In multivariate analysis, the presence of *BRAF* mutations and age at the time of first-line therapy remained the variables significantly associated with a poorer prognosis.

## 1. Introduction

Colorectal cancer (CRC) ranks as the third most frequently diagnosed cancer and the second leading cause of cancer-related deaths, accounting for approximately 1.8 million new cases and 900,000 fatalities annually [1,2]. Both the incidence and mortality of CRC vary significantly across different geographic regions, mainly due to differences in dietary habits, CRC screening implementation and uptake, and broader public health care factors [3]. At the time of initial diagnosis, approximately 15% to 30% of patients present with metastatic CRC (mCRC), while an additional 20–50% of those with locally advanced cancer develop metastases later in the course of their disease; the most common metastatic sites include the regional lymph nodes, liver, lungs, and peritoneum [4,5].

From the pathogenetic point of view, oncogenically activated RAS (mainly KRAS and NRAS) and constitutively mutated BRAF are preponderantly involved in CRC development and progression by inducing cell proliferation through constitutive stimulation of the downstream mitogen-activated protein-kinase (MAPK) pathway [6]. Another CRC pathogenetic mechanism is based on the impairment of the DNA mismatch repair (MMR) genes with subsequent establishment of the microsatellite instability (MSI) phenotype, which has been associated with accumulation of somatic mutations and, in turn, with high antigenic load rendering the tumour more immunogenic [7].

For decades, the therapeutic strategy for the management of mCRC has been constituted by fluoropyrimidine-based chemotherapy regimens, most notably those incorporating 5-fluorouracil (5-FU) and its oral prodrug capecitabine, whose antineoplastic activity is primarily mediated through the inhibition of thymidylate synthase and the consequent disruption of DNA synthesis within malignant cells. These drugs are commonly administered in combination with oxaliplatin or irinotecan to enhance their therapeutic efficacy [8]. Over the past two decades, significant advances have been made in the treatment and prognosis of mCRC patients, including minimally invasive surgical and ablative techniques for the treatment of hepatic and pulmonary metastases, the development of targeted therapies for patients with genetically altered tumours, and the introduction of immunotherapy for patients with mismatch repair (MMR) deficiency or microsatellite instability (MSI) [9,10]. The advent of targeted therapies has extended the overall survival (OS) of patients with mCRC to as much as 30 months [11,12], making molecular profiling before treatment crucial for optimising patient selection and therapeutic decision making. Currently, major scientific societies, such as the National Comprehensive Cancer Network (NCCN) and the Italian Association of Medical Oncologists (AIOM), recommend genetic testing for *KRAS*, *NRAS*, and *BRAF* mutations, as well as assessment of MMR status or MSI, as part of standard clinical practice [13,14].

Despite the undeniable improvements in OS achieved with targeted therapies, their effectiveness remains limited as it is often undermined by the development of resistance, which ultimately leads to disease progression in most patients. This is largely attributed to the complex and still partially understood biology of CRC, as well as to the remarkable ability of cancer cells to adapt, evolve, and finally overcome treatments. Moreover, various factors, such as tumour heterogeneity, disease stage, anatomical location, histopathological characteristics, and occurring molecular alterations, can significantly influence treatment response. Clinical trials typically standardise a wide range of demographic, clinical, pathological, and molecular variables, thereby optimising oncological outcomes. In contrast, real-world outcomes are influenced by additional ‘practical’ factors, including healthcare system policies, access to care, and variations in clinical practice.

For this reason, real-world data on the survival of patients with metastatic CRC and its correlations with several clinicopathological factors are of particular interest. In this retrospective study, we analysed survival outcomes in a cohort of genetically homogeneous Sardinian patients with metastatic CRC at the time of enrolment in a hospital-based manner, examining the relationship between complete molecular profiles—including *KRAS*, *NRAS*, and *BRAF* mutational status along with the MSI assessment, disease stage, and other clinicopathological factors, with the aim of identifying significant correlations that may influence oncological outcomes. Since it is unclear whether the allele frequency (AF) of *RAS* mutations correlates with patient outcome, and the literature is lacking clear evidence on such an issue, we evaluated the prognostic impact of AF in *RAS*-mutated CRC patients.

## 2. Materials and Methods

### 2.1. Patients and Medical Data

Consecutive patients referred for pathological diagnosis and molecular profiling to the Unit of Anatomic Pathology and Histology at the University of Sassari, and the local branch of the Institute for Genetic and Biomolecular Research of the National Research Council (CNR), between 2017 and 2023, were retrospectively included in this study. The inclusion criteria were as follows: (*a*) a confirmed histological diagnosis of colorectal cancer (CRC); (*b*) the ascertained presence of metastatic disease (AJCC stage IV) at the time of inclusion into the study, in accordance with the Italian Association of Medical Oncology (AIOM) guidelines [14]; (*c*) availability of complete molecular profiling data (*KRAS*, *NRAS*, and *BRAF* mutational assessment in addition to the MSI occurrence evaluation); (*d*) availability of complete demographic, clinical, and pathological information; (*e*) three of more years of follow-up from the time of the initial diagnosis; (*f*) age ≥18 years; and (*g*) provision of informed consent to participate in the study.

Medical, pathological, and molecular testing records were collected for all eligible patients. Data regarding histopathological diagnosis, clinical staging, mutational analysis, treatment regimens, and clinical outcomes were extracted and entered into a secure digital database, accessible only to the researchers involved in the study. In our series, all patients were diagnosed with an adenocarcinoma histotype. The study was conducted in accordance with the principles of the latest version of the Declaration of Helsinki and was approved by the Committee for the Ethics of the Research and Bioethics of the National Research Council (CNR n.12629).

### 2.2. Samples and Molecular Testing

Formalin-fixed, paraffin-embedded (FFPE) tissue samples were obtained from the archives of the Anatomic Pathology and Histology of the University of Sassari. All samples underwent morphological re-evaluation, and for each case, eight to ten tissue sections (6 μm thick and containing at least 50% viable neoplastic cells) were prepared. DNA was extracted with the GeneRead DNA FFPE Tissue Kit (QIAGEN, Hilden, Germany) and sequenced with pyrosequencing, which represented the most common sequencing approach for mutation detection used in clinical practice at that time. Testing was conducted in the coding sequence of the following genes: *KRAS* (exons 2, 3, and 4), *NRAS* (exons 2, 3, and 4), *BRAF* (exon 15).

Pyrosequencing was carried out according to the manufacturer’s instructions (Qiagen), and results were elaborated using the PyroMark Q24 2.0 software (Qiagen), including the allele frequency (AF). Concerning this latter feature, the 5% threshold has been reported to better discriminate between patients sensitive and resistant to anti-EGFR drugs [15,16]. In other words, the 5% threshold can be prudentially considered adequate to distinguish patients with *RAS* wild-type (WT) status from those with *RAS* mutations when combined with a good neoplastic cellularity (≥50% tumour cells in the tissue sections undergoing molecular testing) [14,17]. In our series, seven (3.4%) out of 208 patients only presented <5% AF for *RAS* mutations and were classified as *RAS* WT cases. For such cases, AF rate was further confirmed in a duplicated sequencing analysis on FFPE tissue samples macrodissected after comparison with the corresponding haematoxylin and eosin-stained slides, to ascertain that the content of tumour cells from tissue sections was ≥70%.

MSI testing was performed on FFPE primary tumours using the Easy-PGX ready MSI kit (Diatech Pharmacogenetics). This assay is based on multiple real-time PCR reactions analysing eight near-monomorphic microsatellites (BAT-25, BAT-26, NR-21, NR-22, NR-24, NR-27, CAT-25 and MONO-27). According to the revised Bethesda guidelines, the sample is considered to carry microsatellite stability (MSS) if all tested microsatellites present no length changes, MSI low (MSI-L) if one microsatellite is altered, and MSI high (MSI-H) when ≥ 2 altered microsatellites are present.

### 2.3. Statistical Analysis

Patients’ clinicopathological and molecular characteristics are described using absolute frequencies and percentages for categorical variables and mean and median for continuous variables, based on the distribution of the variables. The association between molecular parameters (*RAS* mutations, AF of *RAS* mutations, *BRAF* mutations, and MSI/MSS status) and the main clinicopathological characteristics of patients (gender, age at diagnosis, and primary tumour site) was assessed using Fisher’s exact test for categorical variables and the nonparametric Mann–Whitney or Kruskal–Wallis tests for continuous variables. The AF was analysed as a continuous variable since it is considered a methodologically more correct choice compared to dichotomization, which involves loss of statistical information and produces results that are highly dependent on the chosen cutoff [18], as also done in the context of other malignancies [19]. Overall survival (OS) was estimated using the Kaplan–Meier method, and survival curves were compared between subgroups using the log-rank test. Univariate and multivariate Cox regression models were constructed to estimate the effect of molecular parameters on overall survival. The multivariate model was pre-specified a priori and included mutational status (WT, RAS, BRAF) as the primary exposure variable, with age at first-line therapy and MSI status as adjustment covariates, selected based on their established clinical and biological relevance [20,21]. Statistical significance was set at *p* < 0.05. All statistical analyses were conducted using R (version 4.4.0) within the RStudio environment (version 2026.1.0.392).

## 3. Results

A total of 208 patients with metastatic colorectal cancer (AJCC stage IV), who met all selection criteria indicated in Methods, were enrolled in this study. All patients had available data on anatomical tumour location, tumour stage, histopathological grade, and demographic information (Table 1). Among the patients included, 128 (61.5%) were males and the mean age (±SD) at diagnosis was 65.4 (±10.3) years. Regarding the anatomical site of origin of the tumours, 66 (31.7%) were on the right colon, 45 (21.6%) were on the transverse-left colon, and 92 (44.2%) were in the sigma-rectum. The anatomical site of the tumour was multiple in 5 (2.4%) cases. At the time of diagnosis, the majority (108; 51.9%) of patients already presented with advanced disease (AJCC stage IV); the remaining cases of the series (100; 48.1%) were patients who progressed from lower disease stages (Table 1).

Based on the histopathological characteristics of their tumours and the disease stage, some patients with AJCC stage II (4/27; 14.8%) and most of the patients with AJCC stage III (59/73; 80.8%) received an adjuvant therapy (nearly all cases underwent a treatment based on capecitabine alone or in combination with oxaliplatin). With the exclusion of 16 (7.7%) patients presenting a poor performance status (PS ≥ 3)—who did not receive any pharmacological treatment and were managed exclusively with supportive and palliative care—all 192 (92.3%) patients with advanced disease at the time of enrolment into this study were treated with different types of first-line therapy. A total of 30 (15.6%) patients were treated with chemotherapy alone, specifically CAPOX/FOLFOX in 24 (12.5%) cases and FOLFIRI in six (3.1%) cases. Among the 17 (8.2%) patients positive for MSI-H, the majority of them (13; 76.5%) received first-line immunotherapy with the immune checkpoint inhibitor pembrolizumab. Among the remaining patients, 149 (77.6%) received chemotherapy in combination with targeted biological agents in various regimens. Specifically, 89 patients (59.7%) received CAPOX/FOLFOX/FOLFIRI plus the anti-VEGF agent bevacizumab, 36 (24.2%) received FOLFOX/FOLFIRI plus anti-EGFR agents (cetuximab or panitumumab), 14 (9.4%) received capecitabine plus bevacizumab, and 10 (6.7%) received FOLFOXIRI plus bevacizumab.

### 3.1. Molecular Landscape

Overall, two-thirds (138; 66.3%) of cases harboured at least one mutation in one of the three genes analysed. Among these, *KRAS* mutations were the most frequent, identified in 115 (55.3%) cases, followed by *NRAS* mutations in 8 (3.8%) cases, and *BRAF* mutations at codon 600 in 15 (7.2%) cases (Table 1). Within the *KRAS*-mutated group, mutations were most commonly found at codon 12, occurring in 84 (73.1%) patients, followed by codon 13 in thirteen (11.3%), codon 61 in twelve (10.4%), and codon 146 in six (5.2%) patients. A detailed summary of the specific mutations detected in each gene is provided in Table 2. In our series, no concomitant mutations in *KRAS*, *NRAS*, and *BRAF* genes were detected.

As reported in Table 3, no statistically significant difference in mutation distribution was found between groups according to gender (*p* = 0.143); the median age at first-line of therapy is similar in the WT and *RAS*-mutated groups (66 [60.2–71] and 66 [58.5–74.5] years, respectively), while it is higher in patients with *BRAF* mutations (76 [67.5–78] years) though the difference is not statistically significant (*p* = 0.067). The site of the primary tumour shows a significantly different distribution between the groups according to the mutational status (*p* < 0.001) (Table 3). In WT patients, the tumour is primarily located in the rectosigmoid colon (54.3%), right colon (24.3%), and other sites of the colon (21.4%), with no multisite cases. Among patients with *RAS* mutations, the majority have rectosigmoid (43.1%) or right colon (30.1%) cancer, with a few multisite cases (4.1%). In *BRAF*-mutated patients, the tumour is predominantly located in the right colon (80.0%), while other sites are poorly represented.

In summary, two-thirds (138; 66.3%) of cases harboured at least one mutation in one of the three genes analysed (Table 1). Overall, *KRAS* mutations were the most frequent, identified in 115 (55.3%) cases, followed by *NRAS* mutations in 8 (3.8%) cases, and *BRAF* mutations in 15 (7.3%) cases.

Our CRC population was also classified based on microsatellite instability status, distinguishing patients without instability (MSS; n = 191; 91.8%) from those with a high level of instability (MSI-H, see Methods; n = 17; 8.2%) (Table 4).

Gender distribution showed significant differences between the two groups (*p* = 0.039): among stable patients, the majority (63.9%) were male, while among patients with MSI-H, the majority were female (64.7%) (Table 4). Age at the time of first-line therapy differed significantly between the groups (*p* = 0.002). In patients without microsatellite instability, the median age was 66 years [IQR 59–73], while in MSI-H patients, it was higher, at 76 years [IQR 68–78] (Table 4). The site of the primary tumour also showed significant differences between the groups (*p* = 0.0004). In MSS patients, the most common location is the sigma-rectum (47.1%), followed by the right colon (28.8%) and the remaining colon sites (22.5%), with a few cases of multisite tumours (1.6%). Among MSI patients, the majority of tumours were found in the right colon (64.7%), while other sites were less common (Table 4).

### 3.2. Mutational Status and OS

**Figure 1:**
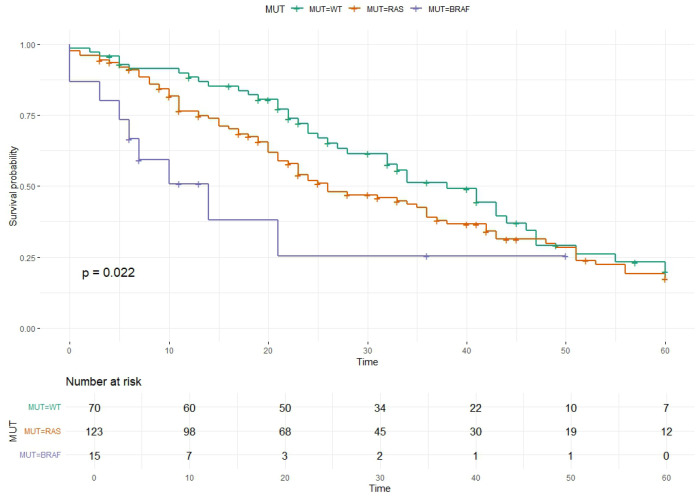
Kaplan–Meier survival curves in accordance with mutation status in the global cohort. MUT = WT, RAS/BRAF wild-type.

Considering the global population, 65.4% deaths were observed in 208 patients with a median follow-up of 34 months (range, 18–74 months). Overall survival (OS) was calculated from the date of first-line therapy or—for the 16 patients with PS ≥ 3 who did not receive any pharmacological treatment (see above and Table 1)—from diagnosis of the metastatic disease to the last control or death date. Median OS was estimated for each group according to the mutational status (WT, *RAS*, and *BRAF*). The median OS for WT patients was 38 (95% CI 32–47) months, with 41 deaths in 70 patients (58.6%). In patients with *RAS* mutations, the median OS was 26 months (95% CI 22–36), with 81 events in 123 patients (65.9%). Finally, in patients with *BRAF* mutations, the median OS was 14 months (95% CI 6–NA), with 9 deaths in 15 patients (60.0%) (Figure 1).

**Figure 2:**
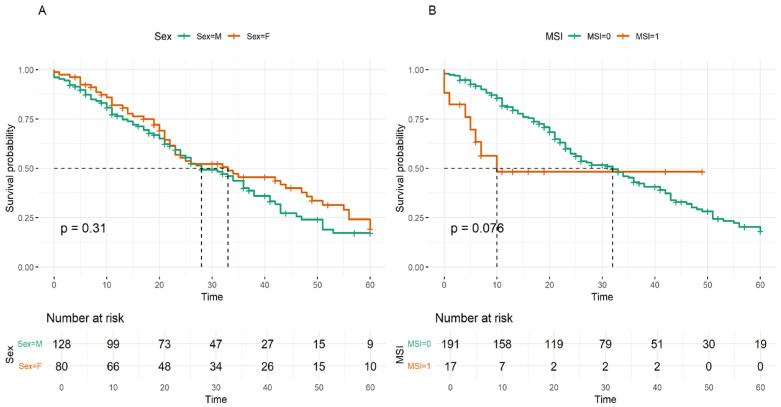
Kaplan–Meier survival curves according to sex (**A**) and MSI status (**B**) in the overall cohort. The black dotted lines indicate the median values. MSI = 0, MSS status; MSI = 1, MSI positivity; F, female; M, male.

Patient sex was not associated with OS (HR 0.81; 95% CI 0.57–1.15; *p* = 0.247) (Figure 2A). Analogously, microsatellite instability status did not show a statistically significant association in the univariate model, although it showed a 90% increased hazard ratio (HR 1.90; 95% CI 0.92–3.91; *p* = 0.082) (Figure 2B).

Univariate Cox regression analysis showed that the occurrence of *BRAF* mutations was associated with a significantly increased hazard of death compared to WT patients (HR 2.69; 95% CI 1.30–5.59; *p* = 0.008) (Table 5). The presence of *RAS* mutations showed a non-significant increased hazard of death (HR 1.30; 95% CI 0.89–1.90; *p* = 0.167). Regarding age at first-line therapy, each 5-year increase in age is associated with a significantly increased hazard of death in univariate analysis (HR 1.12; 95% CI 1.02–1.23; *p* = 0.027) (Table 5). The allelic frequency of *RAS* mutations, centred on 20% and evaluated in 10% increments, was not significantly associated with overall survival (HR 1.05; 95% CI 0.94–1.17; *p* = 0.356) (Table 5). Lack of significant association was confirmed, either including or excluding the seven patients presenting <5% AF for *RAS* mutations.

In multivariate analysis, adjusted for age at first-line therapy and MSI status, results showed that patients with *BRAF* mutations had a more than doubled hazard of death compared to WT patients, with a hazard ratio of 2.25 (95% CI 1.03–4.94; *p* = 0.041). When the primary tumour site was added as a covariate in the multivariate model, the finding remained robust: *BRAF* correlation was significant, with a virtually unchanged HR (2.22; *p* = 0.046). The right colon showed a trend toward a worse prognosis (HR 1.44; *p* = 0.088) but did not reach significance. *RAS* mutations show an increased hazard compared to WT patients, although this association was not statistically significant (HR 1.31; 95% CI 0.90–1.92; *p* = 0.152). Regarding age, each five-year increase at the start of first-line therapy is associated with a 10% increased hazard of death (*p* = 0.031). Finally, MSI status was not significantly associated with overall survival (HR 1.50; 95% CI 0.67–3.30; *p* = 0.308), despite showing a hazard greater than 50% (Table 5).

## 4. Discussion

Targeted therapy and immunotherapy have significantly reshaped the therapeutic landscape of CRC, particularly in the metastatic setting [22]. The selection of targeted agents is driven by the molecular profile of the tumour, with epidermal growth factor receptor (EGFR) inhibitors, cetuximab and panitumumab, demonstrating clinical efficacy exclusively in *RAS* wild-type tumours [11,12,23]. The approval of *KRAS G12C* inhibitors, such as adagrasib combined with cetuximab, exemplifies precision targeting in *KRAS*-mutated CRC, marking a notable therapeutic advance [22]. Conversely, vascular endothelial growth factor (VEGF) pathway blockade with agents such as bevacizumab, aflibercept, and ramucirumab exerts antitumor effects irrespective of *RAS* mutational status, primarily through inhibition of angiogenesis [24]. Additional molecularly targeted approaches, including *BRAF* inhibitors (in combination regimens for *BRAF V600E*-mutant CRC) and *HER2*-directed therapies, have shown efficacy in biomarker-selected subsets [25,26].

Immunotherapy with immune checkpoint inhibitors (ICIs) has emerged as a highly effective strategy in MSI-high (MSI-H) or MMR-deficient (dMMR) CRC, with agents such as pembrolizumab and nivolumab and/or ipilimumab inducing durable responses and prolonged survival [27]. However, most CRC cases present microsatellite stability (MSS) and exhibit limited sensitivity to current immunotherapeutic modalities, necessitating investigation into novel combination strategies to overcome immune resistance [28].

*KRAS* mutations have been estimated to occur in approximately 35–50% of CRC cases, depending on geographic and cohort differences, while *NRAS* mutations are relatively uncommon, occurring in about 4–5% of cases. Finally, *BRAF* mutations, most frequently the *V600E* variant, are observed in roughly 5–7% of tumours. Globally, a mutation in at least one of these genes is expected in 44–62% of the cases. In the present cohort, at least one somatic mutation was identified in two-thirds (66.3%) of analysed cases, with *KRAS* alterations representing the most prevalent event, detected in 55.3% of cases. The prevalence of both *KRAS* and *BRAF* mutations is consistent with that obtained by whole-exome sequencing analysis of either colorectal cancer cell lines or TCGA-analysed primary CRC tissues [29]. Conversely, the frequency of mutated *KRAS* in our series exceeds that reported in other Caucasian cohorts and markedly contrasts with data from earlier investigations conducted in Sardinian populations approximately a decade ago, which documented *KRAS* mutation frequencies ranging from 30% to 36% [30,31,32].

Such discrepancies are likely attributable to substantive methodological refinements implemented across the entire molecular diagnostic workflow, encompassing pre-analytical procedures, such as optimised tissue selection and neoplastic cell enrichment, as well as advancements in sequencing platforms, library preparation protocols, and bioinformatic pipelines for variant detection and interpretation. Moreover, substantial variability in the prevalence of mutations across multiple solid tumour types, not limited to colorectal cancer, has been documented across the Sardinian Island. Notably, populations from the Northern areas of the island exhibit higher mutation rates, particularly involving *RAS* genes, thereby suggesting that the underlying genetic background may modulate the occurrence of oncogenic mutations even within populations that are ostensibly genetically homogeneous [32,33,34].

The prevalence of *RAS* mutations in colorectal cancer within a given population is of critical clinical relevance, as it directly determines the proportion of patients eligible for anti-EGFR therapies. Pivotal randomised trials have conclusively demonstrated that the therapeutic benefit of EGFR inhibition is confined to *RAS* wild-type patients, with these individuals exhibiting significantly higher objective response rates and prolonged OS when cetuximab or panitumumab is combined with standard chemotherapy, a finding subsequently reinforced by pooled analyses and meta-analytic evidence [35,36]. Nevertheless, several large real-world observational studies and datasets have demonstrated that *KRAS* mutational status is not always independently associated with OS, particularly when clinicopathological variables, such as the primary tumour site or the specific *KRAS* variant, are not considered [37,38,39]. For example, Pericay et al. demonstrated that tumour sidedness has a substantial impact on survival among patients with *KRAS* wild-type disease, with left-sided tumours associated with markedly longer overall survival [39]. Similarly, Lavacchi et al. reported significantly prolonged OS in patients harbouring *KRAS* exon 4 mutations compared with those carrying other *KRAS* variants [38].

In our series, analysis of the entire cohort revealed a significant difference in OS between *RAS/BRAF*-mutated and wild-type cases, suggesting that the lack of mutations in these three driver oncogenes is not only predictive of responsiveness to anti-EGFR therapies but is also acting as a favourable prognostic factor. Occurrence of mutations in *BRAF* has widely been reported to act as an unfavourable prognostic factor in CRC patients; analogously, mutations in *KRAS* and *NRAS* genes have been associated with adverse outcomes and propension to metastatic progression [40]. Oncogenic mutations, mostly in *BRAF*, are also significantly associated with worse OS in stage II-III CRC patients [41]. Looking at the other side of the coin, our findings from a genetically homogeneous population like that from Sardinia (in some cases, affecting the tumour molecular background, as previously found by our group [31,32]) evidenced the significantly favourable prognostic role of detecting the *KRAS*/*NRAS*/*BRAF* wild-type status in metastatic CRC patients.

The low prevalence of *NRAS* mutations observed in this study aligns with previous findings, which reported frequencies of 4.1% in Sardinian CRC patients [32]. For *BRAF*, the mutation frequency (7.2%) in our series of patients mostly originating from North Sardinia was higher than that (2.1%) previously reported for patients from the entire island. Again, a geographical variability in the prevalence of mutations across the Sardinian Island may account for such discrepancies, due to the existence of different genetic backgrounds across its different areas that might even modulate the occurrence of oncogenic mutations at the somatic level. Although the small number of cases limits the strength of potential interpretations, a negative impact of *NRAS* and *BRAF* mutations on survival was observed, confirming the widely demonstrated notion that these alterations are typically linked to a more aggressive tumour phenotype and greater resistance to therapies [41,42,43]. Unlike *KRAS*, these mutations activate distinct molecular pathways, potentially leading to different tumour behaviours and therapeutic responses [44,45].

In our series of advanced CRC patients, MSI status showed no association with survival. It has been reported that colorectal cancers carrying the MSI-H status are associated with higher levels of tumour-infiltrating lymphocytes (TILs), which in turn were found to be correlated with better OS in patients with localised CRC [21]. Conversely, the prognostic role of MSI-H or, more generally, of the deficiency in the mismatch repair mechanisms among patients with advanced CRC remains controversial. Currently, MSI-H acts as an effective biomarker for predicting the response to immunotherapy in patients with CRC.

Several studies have compared the prognosis of early-onset and late-onset colorectal cancer, suggesting that tumours arising in younger individuals have pathological features (histotype, degree of differentiation) that have been associated with more aggressiveness, as reported in the SEER database [21]. Although it remains a controversial issue, no differences in overall survival have been reported between these two cohorts; some studies indicate that disease-free survival may only differ between younger and older patients (with early onset associated with worse prognosis) [46,47]. In the white Caucasian population, recent age-stratified analyses have reported no difference in the risk of CRC-specific death among patients with early- or late-onset of the disease [48]. In our study, the age at the time of first-line therapy was found to be significantly associated with a higher risk of mortality, regardless of the treatment regimens patients underwent. This finding is consistent with the literature, indicating comorbidities and other clinical factors (performance status, chronic disease conditions, etc.) may exert an impact on mortality among older patients with CRC [20,49].

Our study has several limitations, chiefly its retrospective design and the relatively small sample size, particularly for cases harbouring *NRAS* or *BRAF* mutations, as well as for those MSI-positive. Another limit of our study is the heterogeneity of the analysed samples for patients with advanced disease, with a mixture of tissue biopsies derived from the most recent metastatic sites and tissue sections from primary tumours, though in different human cancer types, a high consistency for the mutational status of the main driver oncogenes has been demonstrated in paired primary and secondary tumours from the same patients [50,51]. Nevertheless, the study provides novel real-world insights, indicating the complex impact of driver mutations on OS, underscoring the need for further efforts to translate scientific evidence into clinical practice.

## 5. Conclusions

The results of the study showed a higher prevalence of *KRAS* mutations in Sardinian patients with colorectal cancer compared to other Caucasian populations, whereas *NRAS* and *BRAF* mutations were less frequent. Significant differences in OS between *RAS*-*BRAF* mutated and wild-type cases emerged among the advanced CRC patients of our series, with the wild-type tumours associated with a survival advantage. Our findings further highlight that an extensive mutational profiling of the main driver oncogenes is mandatory for more accurately determining the prognosis of patients with advanced CRC. Finally, real-world studies like ours are valuable to clarify how scientific evidence translates into clinical practice within different geographical areas.

## Figures and Tables

**Table 1:** Demographic, clinical, pathological, and molecular features of patients at the time of diagnosis.

Feature	Global Cohort (n = 208)
Age at diagnosis, median ± SD, years	65.4 ± 10.3
Sex, M/F, n (%)	128/80 (61.5/38.5)
AJCC Stage II/III/IV, n (%)	27/73/108 (13/35/52)
T stage 2/3/4, n (%)	9/144/55 (4/69/27)
N stage 0/1/2/3, n (%)	44/97/65/2 (21/47/31/1)
Differentiation grade G1/G2/G3	21/109/78 (10/52/38)
Site of primary tumour: right/transverse/left/sigma-rectum/multiple, n (%)	66/3/42/92/5 (32/1/21/44/2)
Adjuvant treatment, no/yes, n (%)	145/63 (70/30)
First-line treatment, no/yes, n (%)	16/192 (8/92)
Dead, no/yes, n (%)	72/136 (35/65)
*KRAS* status, WT/MT, n (%)	93/115 (45/55)
*NRAS* status, WT/MT, n (%)	200/8 (96/4)
*BRAF* status, WT/MT, n (%)	193/15 (93/7)
MSI-H/MSI-L/MSS, n (%)	17/4/187 (8/2/90)

AJCC: American Joint Committee on Cancer; F: female; M: male; MT: mutated; SD: standard deviation; WT: wild type.

**Table 2:** Distribution of *KRAS* (N = 115), *NRAS* (N = 8), and *BRAF* (N = 15) mutations in the cohort.

Mutations	Variants (%)
**KRAS exon 2**	**97 (84.3)**
G12D	37 (32.2)
G12V	26 (22.6)
G12C	9 (7.8)
G12A	6 (5.2)
G12S	5 (4.3)
G12R	1 (0.9)
G13D	13 (11.3)
**KRAS exon 3**	**12 (10.4)**
Q61H	6 (5.2)
Q61K	2 (1.7)
Q61R	2 (1.7)
Q61E	1 (0.9)
Q61L	1 (0.9)
**KRAS exon 4**	**6 (5.2)**
A146T	5 (4.3)
A146V	1 (0.9)
**NRAS exon 3**	**8 (100)**
Q61K	5 (62.5)
Q61L	1 (12.5)
Q61R	1 (12.5)
Q61H	1 (12.5)
**BRAF exon 15**	**15 (100)**
V600E	15 (100)

**Table 3:** Baseline characteristics of the patient population by mutational status.

Feature	WT (n = 70)	RAS (n = 123)	BRAF (n = 15)	*p*-Value *
Sex	n (%)			
Males	47 (67.1)	75 (61.0)	6 (40.0)	0.143
Females	23 (32.9)	48 (39.0)	9 (60.0)	
Age at first-line therapy				
median [IQR]	66 [60.2–71]	66 [58.5–74.5]	76 [67.5–78]	0.067
Primary tumour site	n (%)			
Right colon	17 (24.3)	37 (30.1)	12 (80.0)	<0.001
Transverse-Left colon	15 (21.4)	28 (22.8)	2 (13.3)	
Sigma-Rectum	38 (54.3)	53 (43.1)	1 (6.7)	
Multiple	0 (0.0)	5 (4.1)	0 (0.0)	

* Sex and tumour site were compared using the χ^2^ test; age at first line therapy using the Kruskal–Wallis test. IQR, interquartile range.

**Table 4:** Baseline characteristics of the patient population by MSI status.

Feature	MSS (n = 191)	MSI-H (n = 17)	*p*-Value *
Sex			
Males	122 (63.9%)	6 (35.3%)	0.039
Females	69 (36.1%)	11 (64.7%)	
Age at first-line therapy			
median [IQR]	66 [59–73]	76 [68–78]	0.002
Primary tumour site			
Right colon	55 (28.8%)	11 (64.7%)	0.0004
Transverse-Left colon	43 (22.5%)	2 (11.8%)	
Sigma-Rectum	90 (47.1%)	2 (11.8%)	
Multiple	3 (1.6%)	2 (11.8%)	

* Sex and tumour site were compared using the χ^2^ test; age at first-line therapy using the Wilcoxon test. IQR, interquartile range.

**Table 5:** Univariate and multivariate Cox regression models showing the hazard ratios for mutational status and the variables investigated in patients.

Variable	Univariate HR (95% CI)		Multivariate HR (95% CI)	
		*p*-Value		*p*-Value
**Wild type**	1.00 (ref)	–	–	–
***RAS* ^mutated^**	1.30 (0.89–1.90)	0.167	1.31 (0.90–1.92)	0.152
***BRAF* ^mutated^**	2.69 (1.30–5.59)	**0.008**	2.25 (1.03–4.94)	**0.041**
AF *RAS*^mutated^ **	1.05 (0.94–1.17)	0.356	–	–
**MSI**	1.90 (0.92–3.91)	0.082	1.50 (0.67–3.30)	0.308
Age at 1st line *	1.12 (1.02–1.23)	**0.027**	1.10 (1.01–1.21)	**0.031**
**Gender**	0.83 (0.58–1.19)	0.313	–	–

Significant correlations are evidenced in bold. * Age at the time of first-line therapy, centred for the median age and with a 5-year increment; ** Allele frequency (AF) centred for 20%, as reference value, for each 10% increment.

## Data Availability

The data presented in this study are available upon request from the corresponding author.

## Abbreviations

The following abbreviations are used in this manuscript:

AF	Allele frequency
CRC	Colorectal cancer
HR	Hazard ratio
MMR	Mismatch repair
MSI	Microsatellite instability
MSS	Microsatellite stability
MT	Mutated
OS	Overall survival
WT	Wild-Type
